# Effects of Ambient Fine Particles PM_2.5_ on Human HaCaT Cells

**DOI:** 10.3390/ijerph14010072

**Published:** 2017-01-12

**Authors:** Qiao Li, Zhihua Kang, Shuo Jiang, Jinzhuo Zhao, Shuxian Yan, Feng Xu, Jinhua Xu

**Affiliations:** 1 Department of Dermatology, Huashan Hospital, Shanghai Medical School, Fudan University, Shanghai 200040, China; dr_liqiao@163.com (Q.L.); yanshuxianhs@sina.com (S.Y.); 2 Department of Laboratory Medicine, Huashan Hospital, Shanghai Medical School, Fudan University, Shanghai 200040, China; kangban@163.com; 3 Department of Environmental Health, School of Public Health and the Key Laboratory of Public Health Safety, Ministry of Education, Fudan University, Shanghai 200032, China; jiangshuofd@sina.com (S.J.); Zhaojinzhuofd@sina.com (J.Z.)

**Keywords:** PM_2.5_, HaCaT cell, skin damage

## Abstract

The current study was conducted to observe the effects of fine particulate matter (PM_2.5_) on human keratinocyte cell line (HaCaT) cells. The potential mechanism linking PM_2.5_ and skin was explored. HaCaT cells were cultured and then accessed in plate with PM_2.5_. Cell viability was tested by Cell Counting Kit-8. The mRNA and protein expression of Filaggrin, Loricrin, Involucrin, and Repetin were analyzed. The levels of Granulocyte-macrophage Colony Stimulating Factor, Thymic Stromal Lymphopoietin, Tumor Necrosis Factor-α, Interleukin-1α, and Interleukin-8 were detected in the supernatant of the HaCaT cell with enzyme-linked immunosorbent assay kits. Cell viability decreased with the increase in PM_2.5_. Compared with the control group, the protein expression of Filaggrin, Repetin, Involucrin, and Loricrin showed different expression patterns in PM_2.5_ treatment groups. The level of Tumor Necrosis Factor-α, Thymic Stromal Lymphopoietin, Interleukin-1α, and Interleukin-8 significantly increased in the cells treated with PM_2.5_. Ambient PM_2.5_ may increase the risk of eczema and other skin diseases. The relative mechanism may be associated with the impairment of the skin barrier and the elevation of inflammatory responses.

## 1. Introduction

Airborne particulate matter (PM) pollution is receiving an increasing amount of attention in China. Particles with an aerodynamic diameter under 2.5 μm (fine particles, PM_2.5_) have become the primary pollutant, which is the most important pollutant when hazes form. In most cities in China, 24 h average concentrations of PM_2.5_ show an obviously high level especially in fall and winter seasons according to the data from Ministry of Environmental Protection of the People’s Republic of China. The Chinese government has established the air quality standard for PM_2.5_ in 2012 [1]. Several experimental and epidemiological studies have demonstrated that PM_2.5_ mainly affect the respiratory system [2] and the cardiovascular system [3], via exerting complex biological effects in these tissues. Recently, the effect of PM_2.5_ on the skin has also been noticed by clinical dermatologists [4,5].

Ambient air pollution was recognized as a crucial risk factor in skin diseases. Previous studies have shown that air pollution may induce or aggravate atopic dermatitis [6,7], which is also associated with an increase in oxidative stress of the skin tissue, the elevation of skin aging, and the impairment of immune function. In addition, one study has found that exposure to ambient PM_2.5_ can aggravate symptoms in children with allergic dermatitis and eczema deterioration [8]. Exposure to high concentrations of PM_2.5_ lead to more pigmented spots in the face and increase the nasolabial folds [9]. However, until now, mechanisms linking ambient PM_2.5_ and skin damage have not been elucidated, and the relevant research is limited.

- The mechanism linking skin damage and environmental factors may have the following aspects.
- Oxidative damage: PM may damage skin through oxidative stress production, which is one of the important causes of skin aging [10].
- Skin barrier function impairment: In the process of keeping skin barrier integrity, several important structural proteins play an important role in the process of skin cell differentiation, such as Loricrin (LOR), Involucrin (IVL), and Filaggrin (FLG) [11].
- Immune injury: Airborne particulate matter may increase keratinocyte inflammatory mediators [12] and cause cutaneous injury in cellular or humoral through immunity and innate immunity pathways. Increasing evidence has shown that the immune injury may play a role in the pathogenesis of various cutaneous inflammatory diseases by releasing cytokines or by expressing membrane-associated adhesion molecules [13,14].

The present study aims to observe the effects of ambient PM_2.5_ on human keratinocyte cell line cells (HaCaT) and explore the potential mechanisms linking PM_2.5_ and skin damage.

## 2. Materials and Methods

### 2.1. PM_2.5_ Sampling

The PM_2.5_ particles were collected using a Thermo Anderson G-2.5 air sampler (Thermo Scientific, Franklin, MA, USA) from May 2014 to December 2014 in a non-industry district in Shanghai, China. Glass microfiber filters (Glass Microfiber Company, Shanghai, China) were used to collect PM_2.5_. After the sampling ended, the filters were cut into small pieces, immerged in three-fold-distilled water, and sonicated for 4 × 30 min with a sonicator (Jeken, Shenzhen, China) for sterilization. The suspension was treated by vacuum-freeze dry, and concentrated fractions were weighted and stored at −20 °C. Before the stimulation, the PM_2.5_ was diluted to 1000 μg/mL and stored at 4 °C.

### 2.2. Cell Culture

HaCaT cells, a spontaneously transformed aneuploid immortal keratinocyte cell line from adult human skin, were purchased from Cells Center of Shanghai Institutes for Biological Sciences (Chinese Academy of Science, Shanghai, China), cultured in defined keratinocyte serum-free medium (K-SFM) (Gibico, Grand Island, NY, USA), and grown at 37 °C in a humidified incubator in a 5% CO_2_ atmosphere. The medium was refreshed every two days, and cells were sub-cultured every four days. Cell culture was performed according to the manufacturer’s manual.

### 2.3. PM_2.5_ Treatment

HaCaT cells were cultured and then accessed in plate with concentrations of 5 × 10^3^ cells/100 μL. After two days of culture, the cells were treated with a series of concentrations (0, 5, 10, 25, 50, 100, 200, 300, 400, 500, and 800 μg/mL) of PM_2.5_ for 24 h to evaluate the concentration-dependent effect. For observing the time-response induced by PM_2.5_, the effects were determined at different exposure times in cells after being treated with PM_2.5_. The morphology of HaCaT cells was observed with a microscope (Nikon, Tokyo, Japan).

### 2.4. Cell Viability Determination

Cell Counting Kit-8 (CCK-8) (Dojindo Laboratories, Tokyo, Japan) is widely used to test cell proliferation and cytotoxicity with high accuracy. In order to observe the cell viability of different concentrations of PM_2.5_, cell viability was determined in HaCaT cells after being treated with 0–800 μg/mL PM_2.5_. Then, the time-response was determined at different exposure times. After treatment with 50 μg/mL PM_2.5_, the HaCaT cells were incubated for 0.5 h, 1 h, 2 h, 3 h, 4 h, 6 h, 8 h, 12 h, 16 h and 24 h, respectively. Enzyme ferment was used to test the cell viability by reading absorbance at 450 nm. The inhibition ratio was calculated and a growth curve was printed. The calculation formula is as follows:

Viability (%) = (Optical Density (OD) control group × OD treatment group) / OD control group 100%

Relative activity (%) = (1 − (test-background) / (control-background)) × 100%.

### 2.5. Western Blot

After treatment with different concentrations (0, 10, 25, 50, and 100 μg/mL) of PM_2.5_ for 24 h, the HaCaT cells were rinsed twice in phosphate buffered saline (PBS). Then, the protein extracts were obtained by cell lysis buffer (Beyotime, Haimen, China) and spun at 14,000× *g* for 10 min at 4 °C. Total proteins for each sample were loaded onto a 10% sodium dodecyl sulfate polyacrylamide gel electrophoresis (SDS-PAGE) gel (Beyotime). After electrophoresis, proteins were transferred onto a nitrocellulose membrane. Blots were rinsed twice in Tris-buffered saline–Tween (TBST). After being blocked for 2 h at room temperature in TBST plus 5% skim milk powder, the nitrocellulose membrane was incubated with different dilutions of primary antibodies—FLG, LOR, IVL, Repetin (RPTN), or β-actin (Abcam, Cambridge, UK)—over 12 h at 4 °C. Then, the membrane was rinsed three times in TBST (10 min each at room temperature) and incubated for 2 h at room temperature with a secondary antibody (Beyotime). Blots were finally rinsed clearly and detected by Immobilon Western (Millipore, Boston, MA, USA). The protein bands were scanned with a LAS3000 imaging system (Fujifilm, Tokyo, Japan), and band density was calculated by Quantity One software (Bio-Rad, Hercules, CA, USA). β-actin was used as a control.

### 2.6. Enzyme-Linked Immunosorbent Assay (ELISA)

The cell supernatant samples were collected from the HaCat cells after treatment with different concentrations (0, 10, 25, 50, and 100 μg/mL) of PM_2.5_ for 24 h. The cytokines Granulocyte-macrophage Colony Stimulating Factor (GM-CSF), Thymic Stromal Lymphopoietin (TSLP), Tumor Necrosis Factor-α (TNF-α), Interleukin-1α (IL-1α), and Interleukin-8 (IL-8) were determined using ELISA kits (AMEKO, Shanghai, China). The tests were performed strictly according to the manufacturer’s instructions.

### 2.7. Statistical Analyses

All analyses were carried out three times independently. The results were presented as means ± standard deviation (SD). The Graphprism 5.0 software (GraphPad Software, San Diego, CA, USA) was used for statistical analysis and graph plotting. The differences among the exposure groups were analyzed using one-way analysis of variance.

## 3. Results

### 3.1. The Morphology of HaCaT Cells

**Figure 1:**
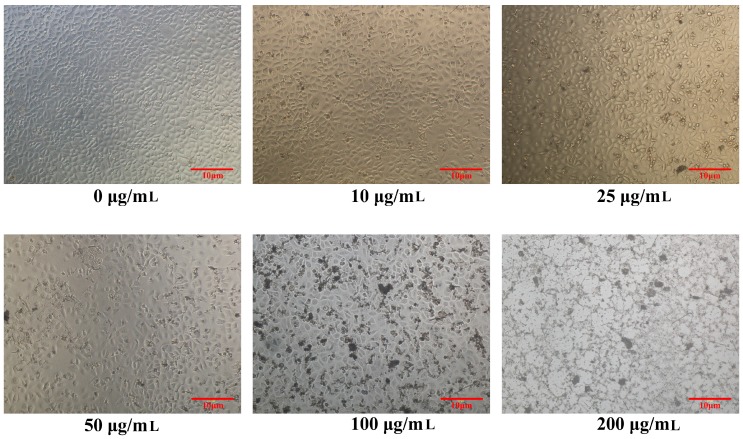
Morphology of human keratinocyte cell line (HaCaT) cells after being exposed to 0, 10, 25, 50, 100, and 200 μg/mL PM_2.5_, respectively. Scale bar: 10 μm.

The morphology of HaCaT cells was observed after being stimulated by different concentrations of PM_2.5_ (Figure 1). When treated with 0 μg/mL PM_2.5_, the HaCaT cells showed a normal shape. However, with the increase in PM_2.5_ concentration, the cell membrane was impaired and the dead cells increased.

### 3.2. Cell Viability Determination

#### 3.2.1. Relationship between PM_2.5_ Concentration and Cell Viability

**Figure 2:**
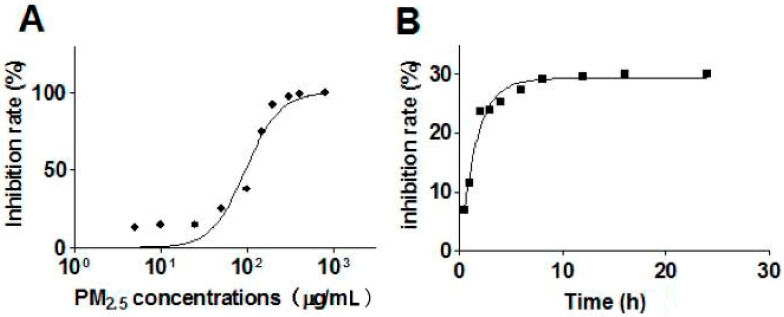
Effects of PM_2.5_ concentrations and exposure times on HaCaT cells viability. (**A**) HaCaT cells were treated with different concentrations of fine particles (PM_2.5_) (from 0 to 800 μg/mL) for 24 h; HaCaT cell viability maintained a low level with lower doses of PM_2.5_, and, when the concentrations of PM_2.5_ exceeded 50 μg/mL, the damage of the cells increased significantly; (**B**) HaCaT cells were treated with 50 μg/mL PM_2.5_, and the CCK-8 assay was used to detect cell viability at 2 h, 3 h, 4 h, 6 h, 8 h, 12 h, 16 h, and 24 h respectively. (Each analysis was carried out three times independently).

The CCK-8 assay was used to detect the cell viability of HaCaT cells after being treated with PM_2.5_. As Figure 2A shows, with the rise in PM_2.5_ concentration, cell viability decreased. At a lower dose of PM_2.5_, the cell inhibition rate maintained a low level. When the concentration of PM_2.5_ exceeded 50 μg/mL, the inhibition rate elevated rapidly. The results indicate that the cytotoxicity of PM_2.5_ significantly increases when the concentration exceeds 50 μg/mL.

#### 3.2.2. Relationship between Exposure Time and Cell Viability

According to the above result, the exposure concentration of PM_2.5_ was set as 50 μg/mL to evaluate the relationship between PM_2.5_ exposure time and HaCaT cells viability. The CCK-8 assay was used to detect cell viability at 2 h, 3 h, 4 h, 6 h, 8 h, 12 h, 16 h, and 24 h, respectively (Figure 2B). When compared with 0 h, a significant reduction of cell viability was observed at 2 h. Nevertheless, from 6 h to 24 h, the cell inhibition rate did not show a significant alteration, which means that the cell viability maintains a stable level (70%) starting from 6 h (Figure 2B).

### 3.3. Western Blot

**Figure 3:**
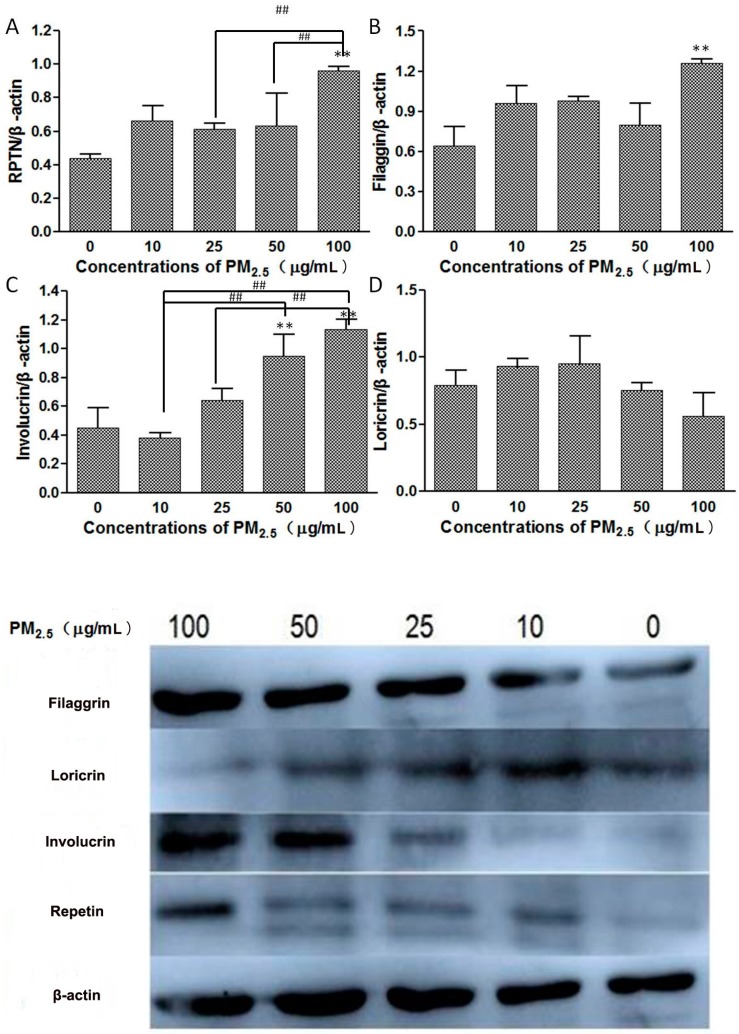
Proteins expression of Repetin (**A**); Filaggrin (**B**); Involucrin (**C**); and Loricrin (**D**) in HaCaT cells after being exposed to different concentrations of PM_2.5_ (0, 10, 25, 50, and 100 μg/mL) for 24 h. ** There were significant differences (*p* < 0.01) between the exposed group and the control. ## There were significant differences (*p* < 0.01) between the exposure groups. (Analysis of variance (ANOVA) statistics was used to calculate the statistical difference between the exposed group and the control and between the four exposed groups. Each analysis was carried out three times independently).

The proteins expression of FLG, IVL, LOR, and RPTN were detected in HaCaT cells by western blot (Figure 3). Increases in IVL and RPTN protein expression in a dose-dependent manner occurred with rises in PM_2.5_ concentration. PM_2.5_ induced a high expression of FLG in cells only in the high PM_2.5_ treatment groups but did not cause significant changes in LOR.

### 3.4. ELISA

**Figure 4:**
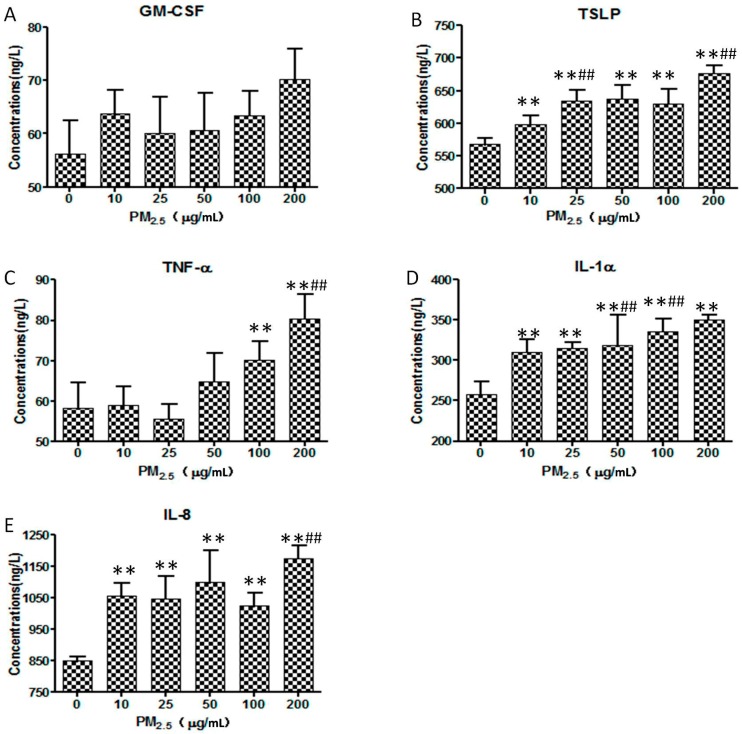
Levels of Granulocyte-macrophage Colony Stimulating Factor (GM-CSF) (**A**); Thymic Stromal Lymphopoietin (TSLP) (**B**); Tumor Necrosis Factor-α (TNF-α) (**C**); Interleukin-1α (IL-1α) (**D**); and Interleukin-8 (IL-8) (**E**) in HaCaT cells after being exposed to fine particles for 24 h. In each figure, the exposed doses from left to right are 0, 10, 25, 50, 100, and 200 μg/mL. ** There were significant differences (*p* < 0.01) between the exposed group and the control. ## There were significant differences (*p* < 0.01) between the group and the former group. (ANOVA statistics was used to calculate the statistical difference between the exposed group and the control and between the exposed group and its adjacent former group. Each analysis was carried out three times independently).

The secretion of GM-CSF, TSLP, TNF-α, IL-1α, and IL-8 were detected in the supernatant of the HaCaT cells (Figure 4). The rise in PM_2.5_ was associated with increased production of the above cytokines, except GM-CSF. The level of TNF-α sharply increased when the concentration of PM_2.5_ exceeded 25 μg/mL. The secretion of IL-8 by HaCaT cells was elevated significantly at low concentrations of PM_2.5_ and maintained a high level with increased concentrations of PM_2.5_. The production of TNF-α, TSLP, and IL-1α increased in a dose-dependent manner, but GM-CSF did not.

## 4. Discussion

Keratinocytes in the skin are the first carrier to be exposed to environmental stresses. The keratinocyte exposed to Asian dust storm particles could upregulate the pro-inflammatory mediators and increase the arylhydrocarbon receptor (AhR) expression, and the latter activation may lead to an increased production of reactive oxygen species [12]. Thus, the current study was aim to observed the influence of ambient PM_2.5_ on HaCaT cells.

A previous study has reported that organic compounds presenting on the surface of PM_2.5_ may penetrate into skin and have direct effects on viable skin cells including keratinocytes [7]. In our study, ambient PM_2.5_ could affect not only the viability of HaCaT cells, but also the expression of skin barrier-related proteins and the synthetic immune-related indicators. Meanwhile, different concentrations of PM_2.5_ induced different responses. With the use of microscope detection and the test of cell activity, the rate of cell damage gradually rose with the rise in PM_2.5_ concentration. When the concentration of PM_2.5_ rose to 50 μg/mL, the cell viability reached a stable level (around 70%). When the concentration of PM_2.5_ exceeded 100 μg/mL, the majority of cells were dead.

The onset of dermatitis is closely related to the reduction of the skin barrier function. Recent studies found that the FLG gene often showed a reduced expression in patients with atopic dermatitis and eczema. An increase in LOR expression, induced by the skin barrier dysfunction was indicated by the immunohistochemistry and Western blot results [15]. IVL is considered to be a human keratinocyte differentiation marker [16]. Previous literature reported that mRNA and protein expression of IVL significantly reduced when the skin barrier function was impaired [15,17,18], yet some studies showed that IVL was upregulated in patients with atopic dermatitis [19,20]. In our study, the results showed that the expression of FLG, IVL, and RPTN elevated significantly after exposure to PM_2.5_, while the expression of LOR showed no change in cells after being stimulated by PM_2.5_. This indicates that the acute exposure of PM_2.5_ might impair the skin barrier by reducing the cell viability directly instead of downregulating the expression of skin barrier-related proteins. On the other hand, the increased levels of FLG, IVL, and RPTN might present a reaction of the skin cells to acute PM_2.5_ exposure. The response of human keratinocytes to chronic and low-concentration PM_2.5_ exposure needs further study.

It has been reported that the immunotoxicity of PM plays an important role in the innate immune system and in cellular or humoral immunity [21]. Ambient PM_2.5_-related skin damage might be caused by activating the innate immune system. TSLP, a member of the cytokine superfamily, plays an important role in the regulation of immune responses by inducing the maturation and activation of multiple immunocytes [22]. TSLP has a high expression in patients with asthma, allergic rhinitis, and atopic dermatitis, which is associated with the relative signal pathway in the pathogenesis of these diseases [14,23,24]. The activation of TSLP could affect innate immunity or the immune response of Th2 cells and activate a nuclear transcription factor (NF-κB) to negatively regulate FLG expression on skin cells [25,26,27]. This study also supports the increased expression of TSLP in HaCaT cells after exposure to PM_2.5_. The results indicate that PM_2.5_ can affect non-specific immunity and cause skin damage.

Additionally, PM_2.5_ can also stimulate human keratinocytes to release a series of cytokines and pro-inflammatory cytokines. Our data showed the levels of TNF-α, IL-1α, and IL-8 significantly increased with the increase in PM_2.5_ concentration, excluding GM-CSF. Pro-inflammatory cytokines further stimulate epithelial cells, fibroblasts, and endothelial cells to secrete cytokines and adhesion molecules (such as IL-8, IL-2, and IL-1). Then, these adhesion molecules and cytokines can accumulate inflammatory cells (such as neutrophils, macrophages, monocytes, and polymorphonuclear leukocytes), resulting in inflammatory response [28,29]. IL-1α was expressed in keratinocytes and was released when the skin was stimulated by external stimulation, which is regarded as an early indicator of skin irritation [30]. However, the mechanisms linking PM_2.5_ or their organic compounds and the expression of inflammation-related cytokines in skin are still limited. Further studies using a suitable experimental system to clarify the mechanisms involved in the modulation of cytokines from keratinocytes by PM_2.5_ pollution are necessary.

## 5. Conclusions

In conclusion, the skin, being an important barrier of the body, can display a series of responses when exposed to external harmful substances. The current study indicates that acute PM_2.5_ exposure can reduce the viability of HaCaT cells and induce the release of inflammatory factors. These results suggest that ambient PM_2.5_ may increase the risk of eczema and other skin disease occurrences dependent on inflammatory activity.
